# Isolation, Identification, and Characterization of Pectinolytic Yeasts for Starter Culture in Coffee Fermentation

**DOI:** 10.3390/microorganisms7100401

**Published:** 2019-09-28

**Authors:** Mesfin Haile, Won Hee Kang

**Affiliations:** Department of Horticulture, Kangwon National University, Chuncheon 24341, Korea; mesfinhaile97@gmail.com

**Keywords:** coffee, enzymes, polygalacturonase, pectin lyase, pectin methylesterase, starter culture

## Abstract

This experiment was carried out to identify and select pectinolytic yeasts that have potential use as a starter culture for coffee fermentation during wet processing. The coffee fruit was fermented for 48 h at 28 °C and a sample was taken from the fermented solution and spread onto yeast extract-peptone-dextrose agar (YPDA) media and incubated at 28 °C. A total of 28 yeasts were isolated, eight of which had the ability to produce pectinase enzymes. The species of those eight yeasts were molecularly identified and confirmed. These yeasts are *Wickerhamomyces anomalus* (strain KNU18Y3), *Saccharomycopsis fibuligera* (strain KNU18Y4), *Papiliotrema flavescens* (strain KNU18Y5 and KNU18Y6)*, Pichia kudriavzevii* (strain KNU18Y7 and KNU18Y8), and *Saccharomyces cerevisiae* (strain KNU18Y12 and KNU18Y13). The pectin degradation index of *S. fibuligera* (strain KNU18Y4), *W. anomalus* (strain KNU18Y3), and *P. flavescens* (strain KNU18Y6) were higher compared to the others, at 178%, 160%, and 152%, respectively. The pectinase enzyme assays were made on two growth media: coffee pulp media (CPM) and synthetic pectin media (SPM). *S. fibuligera* (strain KNU18Y4) and *W. anomalus* (strain KNU18Y3) had great potential in producing polygalacturonase (PG) and pectin lyase (PL) compared to others in both media. However, *S. cerevisiae* strains (KNU18Y12 and KNU18Y13) produced higher pectin methylesterase (PME). Using MEGA 6 software, the phylogenetic trees were constructed to determine the evolutionary relationship of newly identified yeasts from our experiment and previously published yeast species. The sequences of the yeasts were deposited in the National Center for Biotechnology Information (NCBI) database.

## 1. Introduction

Coffee is one of the essential worldwide commodities, next to crude oil. Coffee is the major export commodity of around 60 tropical and subtropical countries [1,2]. Ethiopia is the origin of arabica coffee, and as such, has a long tradition of roasting and drinking this deeply cultural non-alcoholic beverage. Brazil ranks as the leading producer and exporter of arabica coffee, followed by Indonesia, Ethiopia, Philippines, Mexico, Vietnam, and 40 other countries [3]. After harvesting, the coffee fruit is processed using various methods: wet processing, dry processing, and semi-dry processing. During the wet-processing method, the pulp is mechanically separated from fully ripe coffee cherries. Then the fruit is collected and naturally fermented following the process of removal of mucilage by placing it in a fermentation tank and submerging in water for at least 6–24 h [4]

The ultimate goal of fermentation is to remove the mucilage layer, which is made up of complex compounds. Mucilage is a sticky substance and adheres to the coffee pulp, and it comprised around 5% of the coffee cherries in dry matter basis [5]. At the time of maturity of the coffee cherry, calcium pectates found in the middle lamella and protopectin transformed into pectin from the cellular wall [6]. The mesocarp of the coffee cherry is a translucent and gelatinous sweet substance which comprised sugar, minerals and pectin [5]. Silva [7] explained that the microorganisms are responsible for the degradation of the pulp and mucilage (polysaccharides) of the coffee fruit during fermentation. In addition, the production of pectinolytic enzymes and formation of alcohols and acids (butyric acetic, lactic, and other long-chained carboxylic acids) are associated with the microbes [7]. The fermentation processes depend on the growth and metabolic activities of various groups of microorganisms such as yeasts, Gram-negative bacteria, bacilli, filamentous fungi and, lactic acid bacteria and acetic acid bacteria [8]. However, the presence of the microorganism during the fermentation of coffee cherries is relying on the plant variety, processing method, moisture content, the colonizing species enzymatic capacity, the competition of substrates, environmental factors and the microorganism’s antimicrobial activity [9].

The microbial enzymes (pectinases) are responsible for the breaking down of pectin substances. Pectinases are enzymes, which degrade pectin substances and have great importance in the food industry. Reports indicate that microbial pectinases comprised the global food enzymes market by 25% [10]. They hold the first position among the commercially produced industrial enzymes. These enzymes are environmentally friendly and sustainable with potentially wide applications in many industrial activities, such as tea and coffee fermentation, oil extraction, clarification of juice and wines etc. [11]. The main enzymes involved in coffee fermentation are polygalacturonase (PG), pectin lyase (PL), and pectin methylesterase (PME) [12]. These three enzymes have a potential of the complete digestion of pectin to produce galacturonic acid and its oligomers [13,14]. The selection of potential starter culture for coffee fermentation includes production of these enzymes by the microorganisms. The microorganisms used in the production of fermented foods and beverages partly determine their quality. Some of the yeasts that isolated and identified in our study were evaluated for their effect on the coffee quality parameters after fermenting the green coffee beans and found positive results [15].

The search for microbial diversity during coffee processing is required to select microorganisms for starter cultures for the fermentation processes. The isolation and selection of microorganisms for coffee fermentation have significant scope in identifying additional source organisms. The ultimate objectives of this experiment were to isolate, identify, and characterize pectinolytic yeasts. We also evaluated their efficiencies in producing PG, PL, and PME using the synthetic pectin media (SPM) and the coffee pulp-culture media (CPM).

## 2. Materials and Methods

### 2.1. Spontaneous Fermentation and Yeast Isolation

Ripened coffee cherries (20 kg) were harvested from well-managed coffee (Coffea arabica L.) trees that were grown in Chuncheon, Republic of Korea. The coffee cherries were depulped and naturally fermented in 50 mL plastic container at 28 °C without additional starter cultures for 48 h. Various wild yeasts responsible for enhancing spontaneous fermentation activity were expected on the coffee fruit. A sample solution was taken from the fermentation container and serially diluted (10 ^−2^, 10 ^−3^, 10 ^−4^, and 10 ^−5^). Small droplets (30 µL) of the serially diluted solutions were plated onto YPDA (yeast extract-peptone-dextrose agar) media and spread using a glass rod. The cultures in Petri dishes were sealed with Parafilm to prevent contamination of undesired microorganisms. Then the cultures were incubated at 28 °C for four days. The pure culture was used to screen yeast isolates that have distinctive colonial morphologies. They were sub-cultured onto other new YPDA media. The morphological features of the yeast colony that were recorded were color (white and yellow), shape (circular, ovoid), surface (smooth, rough, dry, and powdery), and elevation (flat, raised, convex, pulvinate, and umbonate). The YPDA media was used to culture the yeast from all samples. The media compositions were: dextrose (20 gL ^−1^), yeast extract (5 gL ^−1^), peptone (10 gL ^−1^), and agar (15 gL ^−1^). To suppress the growth of bacteria, 0.1 gL ^−1^ ampicillin powder was added into the media after autoclaving when the solution cooled to 50 °C. All chemicals for this experiment were purchased from Sigma Aldrich LLC (St. Louis, MO, USA).

### 2.2. Pectinolytic Yeast Screening

Pectinolytic activity was assessed using the protocol described by Schwan et al. [16]. The yeasts were grown on plates comprising mineral media: MnSO_4_ (0.05 gL ^−1^), KH_2_PO_4_ (0.2 gL ^−1^), (NH_4_)_2_SO_4_ (1 gL ^−1^), CaCl_2_ (0.05 gL ^−1^), MgSO_4_ (0.8 gL ^−1^); yeast extract (1.0 gL ^−1^); citric pectin (2.0 gL ^−1^); and agar (15 gL^−1^). Enzyme activity was shown by the development of a clear color change (yellow color) around the colonies after flooding the plate with 50 mM potassium iodide–iodine solution.

### 2.3. Pectin Degradation Index (PDI) %

The diameter of the yeast colonies and the pectin degradation zones were measured using a ruler after growing the yeasts on a Petri dish (YPDA + pectin) for five days. The degraded area showed a clear zone after flooding with iodine solution. The clear zone and colony diameters were used to compute the pectin degradation index (PDI), according to the following formula:

Pectin degradation index (PDI) % = (CD + CZ)/CD × 100

where CD is the colony diameter and CZ is the clearing zone diameter.

### 2.4. Molecular Identification and Phylogenetic Tree Analysis of Yeasts

The yeasts were grown on yeast extract-peptone-dextrose (YPD) broth media for 48 h and the yeast cells were collected via centrifugation (10× rpm, 6 min). The yeasts DNA that suitable for use as a template for polymerase chain reaction (PCR) amplification was extracted from approximately 1 × 10^7^ cells. The yeasts were then identified by sequencing the 26S ITS rRNA region of the gene. The 26s rRNA universal primers were used (Table 1). Purified PCR products were sequenced using ABI3730 XL automatic DNA sequencers that were then compared to those available in the GenBank database using the Basic Local Alignment Search Tool (BLAST) algorithm, National Center for Biotechnology Information, MD, USA (NCBI). The phylogenetic tree was made by the neighbor-joining method for this alignment using the Molecular Evolutionary Genetics Analysis (MEGA 6, PSU, USA) software with 1000 bootstrap replications. The final sequences were submitted to the GenBank.

### 2.5. Synthetic Pectin Media and Coffee Pulp Media Preparation for Enzymatic Assays

The SPM was prepared with the following formulation: 10% citric pectin, 0.1% glucose, 0.5% MgSO_4_, 1.0% KH_2_PO_4_, 1.0% (NH_4_)_2_SO_4_, and 0.5% CaCl_2_. The CPM was prepared by measuring 400 g of coffee pulp, including mucilage (*Coffea arabica* L.), and 0.5% glucose. The pulp was boiled using water for 10 min and adjusted to 1 L [17]. Active yeast cells that were grown for 48 h were added with 1.0 × 10 ^4^ cells mL ^−1^ (4.0 Log colony forming unit (CFU) mL ^−1^). The cultures were incubated at 28 °C for 96 h at 120 rpm and periodically sampled at 24, 48, 72, and 96 h. The supernatants were collected for PG, PL, and PME activity from both SPM and CPM growing conditions.

In addition, the fermentation characteristics of the CPM culture, such as yeast cells, pH, and °Brix, were regularly measured at 24-h intervals. Serial dilutions were prepared from the fermented solution and plated onto YPDA media. The living yeast colonies were counted using the Neubauer hemocytometer slide (Electron Microscopy Sciences, PA, USA) and expressed in Log CFU value. A pH meter was used to measure the pH of the fermented solution. A refractometer (ATAGO Pocket Refractometer, Tokyo, Japan) was used to estimate the °Brix of the media. All the above measurements were made in three replications regularly at 24-h intervals.

The protein content of the coffee pulp used to make the CPM was analyzed using the protocol mentioned by Bradford [18], and bovine serum albumin (BSA) was used as a standard. The total sugars [19] and soluble pectin [20] were determined.

### 2.6. Enzyme Assays

#### 2.6.1. Pectin Lyase Activity

The pectin lyase activity was estimated in the culture using the method described by Pitt [21] as modified by Kashyap et al. [22]. The reaction mixture consisted of 5 mL of 1.0% (*w v* ^−1^) citrus pectin (85% esterified) in 0.5 M Tris–HCL, pH 6.8, and 1.0 mL of culture supernatant. The reaction mixture was incubated for 2 h at 40 °C. Then, 0.6 mL of 9% zinc sulfate and 0.6 mL of 0.5 M sodium hydroxide were added to discontinue the reaction. After that, using a centrifuge (6000 *g* for 5 min.), 5 mL of supernatant was collected from the mixture and combined with 3 mL of 0.04 M thiobarbituric acid, 2.5 mL of 0.1 M HCl, and 0.5 mL of distilled water. This mixture was boiled for 30 min then cooled to room temperature before reading the absorbance at 550 nm using a spectrophotometer. One unit of enzyme activity (U mL ^−1^) of pectin lyase increased the absorbance by 0.01 units.

#### 2.6.2. Polygalacturonase Activity

The polygalacturonase activity was examined according to the protocol defined by Schwan et al. [16]. A measurement of 0.1% polygalacturonic acid (*w v* ^−1^) was added in 0.1 M citrate buffer, and the pH was adjusted to 5.0. Then, the reaction mixture was prepared using 1 mL of PG from the first buffer mixed with 1.5 mL of sample supernatant; this was followed by incubation for 1 h at 40 °C. The reaction was stopped by the addition of 1.5 mL of DNS [23]. The mixture was boiled for 5 min and then cooled in an ice bath. The absorbance reading was made at 600 nm using a spectrophotometer and measured with a proper calibration curve. One unit of enzyme activity (U mL ^−1^) was expressed as 1 mol of galacturonic acid liberated mL ^−1^ min.

#### 2.6.3. Pectin Methylesterase

Pectin methylesterase was measured using the protocol introduced by Baracat et al. [24] with the continuous titrimetric determination of the carboxyl groups liberated from methyl ester bonds. The reaction was carried out with 3 mL of the enzymatic micro solution, to which 20 mL of 1% Sigma citric pectin in NaCl 0.1 M pH 7.5 solution was added. The mixture was incubated for 30 min keeping the pH at 7.5 by the addition of NaOH 0.02 M. The absorbance reading was made at 550 nm using a spectrophotometer. PME activity was expressed as the micro equivalents of polygalacturonic acid produced mL ^−1^ h ^−1^.

### 2.7. Statistical Analysis 

Analysis of variance (ANOVA) was computed for testing the significances of the experiment. Data were summarized in an Excel program and analyzed using SAS statistical software (SAS Institute, Cary, NC, USA). Mean separation was done using Fisher’s Least Significant Difference.

## 3. Results

### 3.1. Isolation, Selection, and Morphological Characterization of Yeasts

The fermentation of coffee cherries was done for 48 h with yeasts and other microorganisms that naturally existed on the coffee fruit. The samples were taken and spread on YPDA media to isolate the yeasts. A total of 28 yeasts were isolated during coffee fermentation using the wet-processing method. Among the isolated yeasts, eight of them showed pectinolytic activity after testing them on YPDA media enriched with citrus pectin. The isolated yeasts colonies were morphologically characterized according to their shape, color, elevation surface, and margin (Table 2).

### 3.2. Pectin Degradation Index (PDI) %

As shown in Table 3, the maximum pectin degradation index was derived after five days of incubation on YPDA media that has a citrus pectin. The PDI% of yeasts was ranged from 110 to 178%. The highest PDI was obtained from *S. fibuligera* (strain KNU18Y4) at 178%. The PDIs of *W. anomalus* (strain KNU18Y3) and *P. flavescens* (strain KNU18Y6) were 160% and 152%, respectively (Table 3). However, relatively, the lowest PDI % was obtained from *P. kudriavzevii* KNU18Y7, and it was 110%. The PDI of the *S. cerevisiae* KNU18Y12 and *S. cerevisiae* KNU18Y13 was 121% and 118%, respectively.

#### Molecular Identification and Phylogenetic Trees

**Figure 1:**
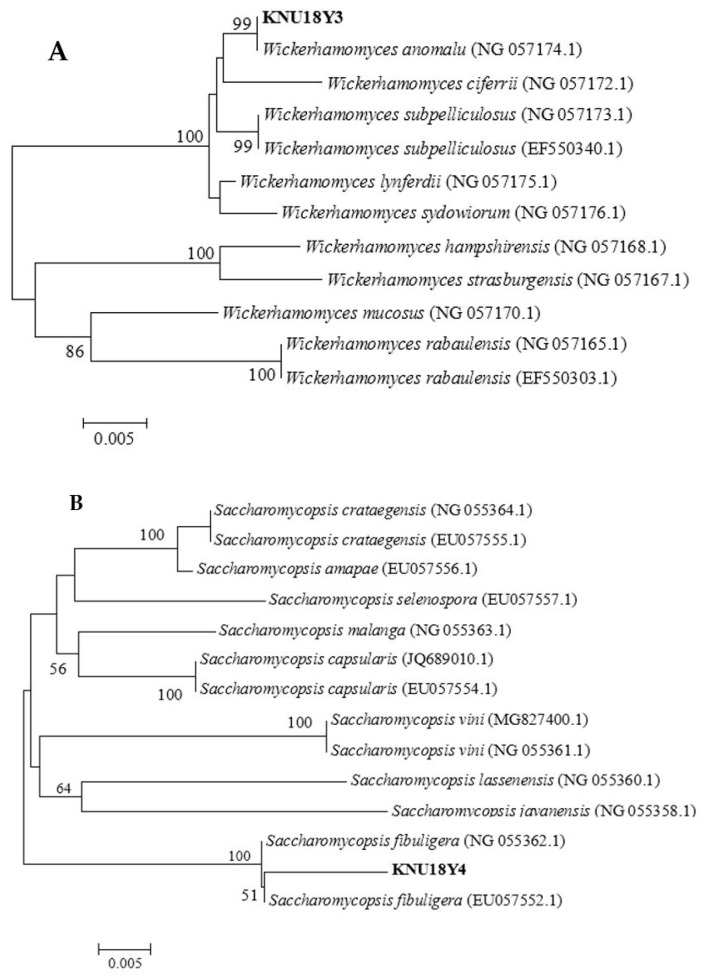

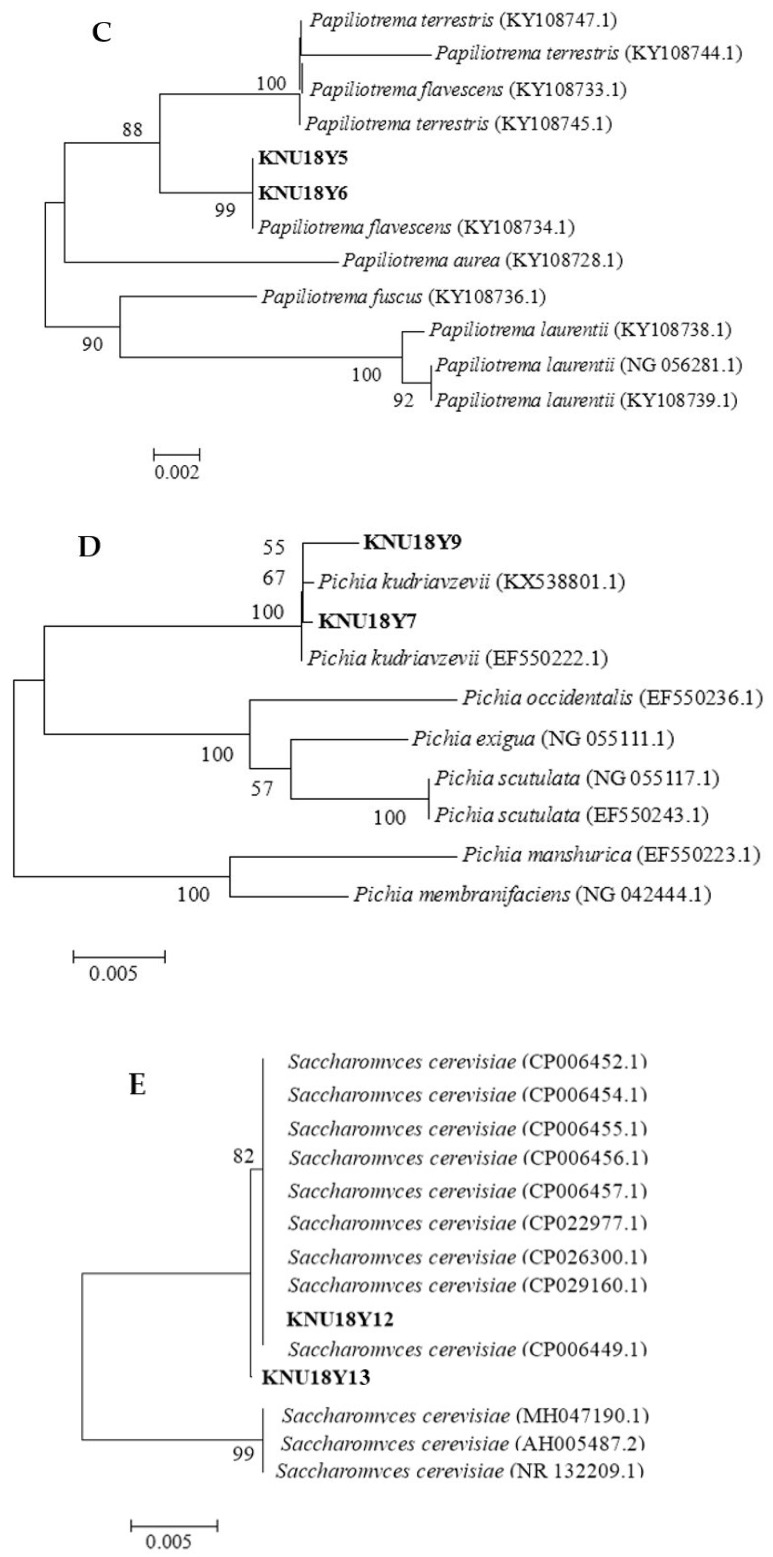
Phylogenetic relationship between the identified yeasts and other 26S rRNA sequences of published strains. (**A**) *Wickerhamomyces anomalus* strain KNU18Y3, (**B**) *Saccharomycopsis fibuligera* strain KNU18Y4, (**C**) *Papiliotrema flavescens* (strain KNU18Y5, KNU18Y6), (**D**) *Pichia kudriavzevii* (strain KNU18Y7 and KNU18Y9) and (**E**) *Saccharomyces cerevisiae* (strain KNU18Y12 and KNU18Y13).

Based on the preliminary pectinolytic yeasts screening results, eight isolates were selected and identified molecularly by sequencing the 26s rRNA gene (D1/D2 region) using universal primers (Table 1). The phylogenetic trees were constructed using MEGA 6 software to reveal the evolutionary distance between the yeasts, which were identified from our study and previously reported yeast species in the National Center for Biotechnology Information (NCBI) database (Figure 1A–E). The identified yeasts were submitted to the GenBank with the accession numbers mentioned in Table 4.

### 3.3. Fermentation Characteristics

The living yeast cells were counted at every 24-h interval. A significant difference (*p* < 0.05) in living yeast cells were observed among the evaluated yeasts at different fermentation hours (Table 5). An increasing trend in living yeasts cell was observed from the initial inoculation up to 48 h of fermentation. Then, decreasing growth trends were observed in all yeasts at 72 and 96 h of fermentation. The maximum living yeast cell growth was found from all yeasts at 48-h fermentation. The number of living yeast cells of *S. cerevisiae* KNU18Y13 were significantly higher (8.23 Log CFU mL^−1^) compared to other yeasts at 48 h fermentation. Among the tested yeasts, *S. cerevisiae* strain KNU18Y13 had a unique characteristic in terms of growth habit. It is a highly reproducible yeast compared to other yeasts and can ferment very quickly, with a noticeably high production of carbon dioxide. From the experimented yeasts, *S. fibuligera* showed low reproduction rates (at each fermentation period compared to others (Table 5)). There was no significant difference (*p* > 0.05) in living yeast cells at 72 and 96 h of fermentation among *W. anomalus* KNU18Y3, *P. flavescens* KNU18Y6, and *S. cerevisiae* KNU18Y13.

The protein content, the total sugar, and total pectin in the coffee pulp that was used to make the CPM were measured as dry matter, accounting for 14.30%, 12.45%, and 6.70%, respectively. The pH of fermented solutions was regularly monitored and recorded. The initial (0 h) pH of the CPM was adjusted at 5.72. The pH of the CPM continuously dropped starting from the initial hours of fermentation until 72 h and began increasing at 96 h in all yeasts except the *S. fibuligera* KNU18Y4 inoculated media. Comparatively, the pH of the CPM with the *S. fibuligera* KNU18Y4 continuously declined from the 24-h period to 96 h at a slow rate. In the *P. flavescens* KNU18Y5 inoculated treatment, the pH decreased from the initial fermentation until 48 h and began increasing at 72 and 96 h. During the first 24-h fermentation, the pH decreased at a higher rate compared to other fermentation hours.

The °Brix of the CPM was measured from beginning to end of the fermentation process. The initial °Brix of the CPM was 5.4. There were significant differences among yeasts in reducing the °Brix contents during each fermentation period. The °Brix was continuously dropped in all fermentation treatments (Table 6). During the first 24 h of fermentation, the °Brix decreased at a higher rate compared to the other fermentation periods. The *W. anomalus* KNU18Y3, *S. fibuligera* KNU18Y4, *P. flavescens* KNU18Y6, and *S. cerevisiae* (KNU18Y12 and KNU18Y13) strains inoculated CPM °Brix was declined below 1% at the end of 96 h fermentation. After 96 h fermentation, the °Brix of CPM was changed from 5.4 to nearly 1.0% in *P. flavescens KNU18Y5 and P. kudriavzevii* strains (KNU18Y7 and KNU18Y9). The lowest °Brix was recorded from *W. anomalus* KNU18Y3 inoculated CPM (0.40%) at the same time the pH was lower (4.75) compared to other treatments after 96 h fermentation.

### 3.4. Enzyme Assays

#### 3.4.1. Pectin Lyase

The PL activity of different yeasts was determined at different fermentation hours in both CPM and SPM growth conditions. The PL activity of *S. fibuligera* KNUY18Y4 and *W. anomalus* KNU18Y3 were significantly (*p* < 0.05) higher compared to other yeasts during all fermentation periods in both CPM and SPM growth conditions (Table 7). The maximum PL activity was found during the 24-h fermentation from all yeasts in both media. Regarding the fermentation hours, the highest PL activity of *S. fibuligera* KNUY18Y4 obtained at 24 h fermentation and it was 17.66 and 117.55 U mL ^−1^. Similarly, the highest PL activity of *W. anomalus* KNU18Y3 was 16.96 U mL^−1^ in CPM and 16.83 U mL ^−1^ in SPM condition. The PL activity showed a decreasing trend as the fermentation hours progressed from 24 to 96. The PL activity of the yeasts showed similar patterns in both media, and the results were approximate. In both media conditions, the lowest PL activity was obtained from *S. cerevisiae* KNU18Y12, *P. kudriavzevii* KNU18Y9, *S. cerevisiae* KNU18Y13, and *S. cerevisiae* KNU18Y12 during the 24, 48, 72, and 96-h fermentation periods, respectively. The PL activity between *P. flavescens* strains (KNU18Y5 and KNU18Y6), did not show a remarkable difference at 24 h in the CPM condition and it was 16.16 and 16.42 U mL ^−1^, respectively (Table 7). However, at 48, 72, and 96 h, a significant difference between *P. flavescens* strains was observed in CPM and at 72 h in SPM growth condition. The *P. kudriavzevii* KNU18Y7 produce significantly higher at 48 and 96 h compared to *P. kudriavzevii* KNU18Y9 in both media types. In CPM condition, the PL activity of *P. kudriavzevii* KNU18Y7 at 48 and 96 h was 14.57 and 7.81 U mL ^−1^, respectively. However, in SPM condition, the PL activity of *P. kudriavzevii* KNU18Y7 was 14.51 and 7.76 U mL ^−1^ at 48, and 96 h. Generally, the PL activity of all yeasts was approximately increased by double at 24 and 48 h compared to 72 and 96 h durations in both media conditions.

#### 3.4.2. Polygalacturonase Activity

The PG activity of eight yeasts was evaluated at different fermentation hours by growing them in CPM and SPM conditions. Like PL, the PG activity of *S. fibuligera* KNUY18Y4 and *W. anomalus* KNU18Y3 were significantly (*p* < 0.05) higher compared to the other yeasts at 24, 48, and 72 h fermentation, respectively, in both CPM and SPM (Table 8). The highest PG activity of *S. fibuligera* KNUY18Y4 was found at 48 h fermentation and it was 8.28 and 8.21 U mL ^−1^ in CPM and SPM condition, respectively. However, relatively, the maximum PG activity of *W. anomalus* KNU18Y3 was obtained at 24 h fermentation and it accounts 8.01 U mL ^−1^ in CPM and 7.98 U mL ^−1^ in SPM. Regarding the fermentation time, the highest PG activity was found at 24 h from all yeasts except *S. fibuligera* KNUY18Y4 in both media. At 24-h fermentation, the lowest PG was secreted by *S. cerevisiae* strain KNU18Y13 and it was 6.06 U mL ^−1^ in CPM and 6.03 U mL^−1^ in SPM conditions. We found a significant difference between the two strains of *P. flavescens* (KNU18Y5 andKNU18Y6) in producing PG at 48 and 96 h, respectively, in both media, and at 24 h in the SPM condition. However, at 72 h fermentation in both media and 24 h fermentation in the CPM condition, these two strains of *P. flavescens* did not show significant variation in producing the PG enzyme (Table 8). Like *P. flavescens* strains, a similar result trend was observed between the *P. kudriavzevii* strains (KNU18Y7 and KNU18Y9). Regardless of yeasts, the highest activity of *P. kudriavzevii* KNU18Y9 (6.98 and 6.67 U mL ^−1^) and *P. kudriavzevii* KNU18Y7 (6.54 and 6.41 U mL ^−1^) was found at 24-h fermentation in both media types (Table 8). Generally, apart from *S. fibuligera* KNUY18Y4 at 48 h, the PG activity showed a decreasing trend as the fermentation hours exceeded from 24 to 96.

#### 3.4.3. Pectin Methylesterase

There were significant (*p* < 0.05) differences among the PME activity of yeasts at different fermentation hours in both the CPM and SPM growth conditions (Table 9). During 24 h fermentation, the PME activity was not significantly different among the tested yeasts, except for the *S. cerevisiae* strains, in both media types. PME activity of all yeasts showed an increasing trend as the fermentation proceeded from 24 to 48 h. However, a decreasing trend observed at 72 and 96 h fermentation in both media conditions. The highest PME activity throughout the fermentation period was obtained from all yeasts at 48 h fermentation in both media types. Comparatively, a significant PME activity found from *S. cerevisiae* strains (KNU18Y12 and KNU18Y13) in both media. The maximum PME activity of *S. cerevisiae* strains (KNU18Y12 and KNU18Y13) was 18.25 and 16.02 U mL^−1^ in CPM and it was 18.01 and 17.63 U mL^−1^ in SPM condition, respectively (Table 9). Unlike PL and PG, the PME activity of *S. fibuligera* KNU18Y4 and *W. anomalus* KNU18Y3 yeasts was lower in both media types compared to other yeasts. At 48 h, the PME activity of *W. anomalus* KNU18Y3 was 15.68 and 15.56 U mL ^−1^ in CPM and SPM condition, respectively. However, the *S. fibuligera* KNU18Y4 PME activity was higher at 48 h fermentation compared to other fermentation hour treatments and it was 15.96 U mL ^−1^ in the CPM condition, and 15.72 U mL ^−1^ in the SPM condition. The PME activity did not show a significant difference between *P. flavescens* strains KNU18Y6 and KNU18Y5 at each fermentation period in both media (Table 9). Likewise, the PME activity was not significantly different between the *P. kudriavzevii* strains (KNU18Y7 and KNU18Y9) in both media at 24, 48, 72, and 96 h of fermentation.

## 4. Discussion

Fermentation is an important step during coffee processing to remove the mucilage (sticky polysaccharide substance) form the parchment coffee. However, fermentation in wet processing is critical because it creates a diverse aroma and flavor beyond its natural flavor and taste. Pectinase enzyme accelerates tea fermentation by breaking down the pectin substance which presents in the tea leaves [25]. Pectinase enzyme-producing yeasts are responsible for speeding up the fermentation process and removing the pectin substances during coffee processing. For these reasons, isolation, identification, and characterization of yeasts that can produce pectinase enzymes (pectin lyase, polygalacturonase, and pectin methyl esterase) are essential. This process needs frequent study to develop starter cultures for coffee fermentation. A microbiological culture that facilitates fermentation activities is called starter culture. Starter cultures are widely used in the food industry to prepare such products as yogurt, wine, and beer [26]. In controlled coffee fermentation, starter culture may improve the quality of coffee and increase its economic value, thereby enhancing the incomes of farmers [27]. Yeast populations increased as the fermentation duration increased from 0 to 96 h. However, the rate of yeast growth showed a sharper increase during the first 48 h, a result supported by Kamassah et al. [28]. The growth of the yeasts decreased and approximately leveled out to a steady growth at 72 and 96 h compared to the first 48 h. The decrease after 48 h might be associated with pH changes (Table 6) and depletion of the substrates, such as the total soluble solid content (Table 6). The change in pH occurred during fermentation of CPM with different yeasts. The decreases in pH during fermentation are associated with the production of acids. It was found that the production of organic acid and absorption of amino acids have significant effects. Pectinolysis enabled reduction in demucilization time which was evident with reduction in pH value [29]. As the yeasts grew in the CPM for 96 h, the °Brix was substantially reduced. However, the ability to decrease the °Brix content was significantly varied among species as well as within strains (Table 6).

Pectinase enzyme is produced by several microorganisms. It was reported in bacteria such as *Xanthomonas* sp., *Bacillus* sp. and in very few yeasts such as *S. cerevisiae* and *Candida boidinii* [30]. Pectinase activity was found in yeasts and yeast-like microorganism such as *Dioszegia* sp., *Phenoliferia glacialis* and *Tetracladium* sp. isolates and the enzyme was identified as polygalacturonase [31]. In our experiment, the PL, PG, and PME activities of yeasts decreased as the fermentation hours increased from all yeasts. A similar result reported by Oumar et al., [32] the PL activity of *Bacillus subtilis strain Btk 27* was reduced as the incubation time extended more than 48 h. The pectinase enzyme production among yeasts was different. This indicated that the ability of the microorganism producing the pectinase enzyme varied among yeasts species and strains. We have identified a pectinase enzyme-producing *S. cerevisiae* (KNU18Y12 and KNU18Y13) during coffee fermentation by wet processing. Furthermore, we have identified two *pectinolytic P. kudriavzevii* yeast strains. Koffi et al. [33] isolated and identified *S. cerevisiae YB14* and *P. kudriavzevii YP13* yeasts that were able to produce the pectinase enzyme during cacao fermentation. However, pectinolytic *P. kudriavzevii* yeast was examined and showed a potential ability in producing PG enzymes during cacao mucilage fermentation [34]. In this experiment, a potential pectinase enzyme-producing yeast, *W. anomalus,* was identified during a wet-processing method. The PL and PG activity *W. anomalus* in our experiment was significantly higher than other examined yeasts following *S. fibuligera* KNU18Y4. Martos et al. [35] reported a potential pectinase enzyme-producing *W. anomalus* from citrus peel. They also mentioned its maximal PG activity was at pH 4.5. However, in our experiment, we did not adjust the pH of the medium during fermentation activity, rather the pH and the enzyme activity was measured at 24 h interval. The pH was 4.93 after 24-h fermentation in *W. anomalus* inoculated media and the PG activity was higher compared to the rest other fermentation durations (48, 72, and 96). The PG activity of yeasts produced in an optimum pH in the acidic region between 3.5 and 5.5 [35]. The *W. anomalus* is grouped as a biosafety level 1 organism that can grow under severe environmental stress conditions, such as high and low pH, high osmotic pressure, anaerobic conditions, and low water activity [36]. A study showed that as the pH increased from 3 to 4, the *Aspergilus niger* pectinase enzyme production also increased [37].

To the best of our knowledge, the other three yeasts *S. fibuligera* KNU18Y4 and two *P. flavescens* (KNU18Y5 and KNU18Y6) are being reported for the first time as pectinase enzyme-producing yeasts that are identified during coffee fermentation in the wet-processing method. The production of pectinase enzymes varies among the identified yeasts in our experiment. These variations might be associated with the difference of microorganism population (Table 5) and pH condition (Table 6) during the fermentation period. Pectinase production by fungi varies according to the type of strain and cultivation conditions (initial pH, inoculum size, and incubation period) [38]. Regarding the pectin lyase activity, the first 24-h fermentation period produced a greater amount in all tested yeasts in both media types. Besides the yeasts’ ability to produce the pectinase enzyme, we have evaluated the impact of some of these yeasts by fermenting green coffee beans to improve the antioxidant activity, total polyphenol, flavonoid, and tannin contents [15]. They showed positive results in improving the flavonoid, polyphenol, and antioxidant activity [15].

## 5. Conclusions

In this study, we have isolated, identified, and characterized eight pectinase enzyme-producing yeasts during wet-processing methods of fermenting coffee. Out of the identified yeast, *S. fibuligera* KNU18Y4 and *W. anomalus* KNU18Y3 have the prominent capability of producing the PG and PL enzymes in both the coffee pulp media and synthetic pectin media. The two *S. cerevisiae* strains (KNU18Y12 and KNU18Y13) produce a higher PME. We have also identified two strains of *P. flavescens* (KNU18Y5 and KNU18Y6) and *S. fibuligera* KNU18Y4 that have not previously been reported as pectinase enzyme-producing yeast. Regarding the fermentation duration, the 24 and 48 h cultivation of the tested yeasts produced a higher PL enzyme in both media types. Comparatively, the PG and PME activity of all yeasts was high at 24 and 48 compared to 72 and 96 h fermentation. Generally, these yeasts have the potential to be used as a starter culture during fermentation of coffee. Additionally, these yeasts can also be used in the food industry for pectinase enzyme production. Isolation and identification of yeasts to develop starter cultures for coffee fermentation are relevant and should be continued in the future.

## Figures and Tables

**Table 1:** Details of the primers used in the present study.

Primer Name	Sequence	Type
Forward	5’-ACCCGCTGAACTTAAGC -3’	Universal
Reverse	5’ -TACTACCACCAAGATCT -3’	Universal

**Table 2:** Morphological features of the eight yeast colonies.

Yeasts	Shape	Color	Elevation	Surface	Margin
*W. anomalus* KNU18Y3	Circular	White	Raised	Smooth	Entire
*S. fibuligera* KNU18Y4	Circular	White	Convex	Rough	Entire
*P. flavescens* KNU18Y5	Circular	Yellow	Pulvinate	Rough	Entire
*P. flavescens* KNU18Y6	Circular	Yellow	Pulvinate	Smooth	Entire
*P. kudriavzevii* KNU18Y7	Circular	White	Umbonate	Smooth	Entire
*P. kudriavzevii* KNU18Y9	Circular	White	Pulvinate	Smooth	Entire
*S. cerevisiae* KNU18Y12	Circular	White	Convex	Smooth	Entire
*S. cerevisiae* KNU18Y13	Circular	White	Pulvinate	Smooth	Entire

**Table 3:** Pectin degradation index after 5 days incubation on YPDA media that supplemented a citrus pectin.

Yeasts	PDI %
*W. anomalus* KNU18Y3	160^b^ ± 1.73
*S. fibuligera* KNU18Y4	178^a^ ± 4.04
*P. flavescens* KNU18Y5	129^c^ ± 4.62
*P. flavescens* KNU18Y6	152^b^ ± 4.04
*P. kudriavzevii* KNU18Y7	110^d^ ± 2.89
*P. kudriavzevii* KNU18Y9	125^c^ ± 4.04
*S. cerevisiae* KNU18Y12	121^cd^ ± 2.31
*S. cerevisiae* KNU18Y13	118^cd^ ± 4.62

Results are presented as mean ± standard deviation (*n* = 5). Means denoted with different letters within column are significantly different.

**Table 4:** The accession number of the identified yeasts species and strain code.

Accession Number	Yeast Species Name	Strain Code
MH483547	*Wickerhamomyces anomalus*	KNU18Y3
MH483929	*Saccharomycopsis fibuligera*	KNU18Y4
MH484046	*Papiliotrema flavescens*	KNU18Y5
MH485393	*Papiliotrema flavescens*	KNU18Y6
MH488970	*Pichia kudriavzevii*	KNU18Y7
MH487468	*Pichia kudriavzevii*	KNU18Y9
MH491423	*Saccharomyces cerevisiae*	KNU18Y12
MH488975	*Saccharomyces cerevisiae*	KNU18Y13

**Table 5:** The living cells of yeast species at different fermentation hours (24, 48, 72, and 96) in CPM.

Yeasts	Log CFU mL^−1^			
	24 h	48 h	72 h	96 h
*W. anomalus* KNU18Y3	7.08^f^ ± 0.23	8.21^b^ ± 0.32	7.22^a^ ± 0.52	7.23^a^ ± 0.10
*S. fibuligera* KNU18Y4	6.80^g^ ± 0.11	8.04^e^ ± 0.52	7.11^d^ ± 0.41	7.12^c^ ± 0.08
*P. flavescens* KNU18Y5	7.10^e^ ± 0.21	8.17^d^ ± 0.46	7.16^b^ ± 0.36	7.15^b^ ± 0.15
*P. flavescens* KNU18Y6	7.13^d^ ±0.14	8.20^b^ ± 0.26	7.23^a^ ± 0.21	7.22^a^ ± 0.17
*P. kudriavzevii* KNU18Y7	7.17^c^ ±0.12	8.19^c^ ± 0.13	7.15^c^ ± 0.25	7.15^bc^ ± 0.20
*P. kudriavzevii* KNU18Y9	7.13^d^ ± 0.15	8.18^cd^ ± 0.26	7.16^b^ ±0.14	7.17^b^ ± 0.16
*S. cerevisiae* KNU18Y12	7.19^b^ ± 0.09	8.18^cd^ ± 0.18	7.17^b^ ± 0.11	7.16^b^ ± 0.19
*S. cerevisiae* KNU18Y13	7.22^a^ ± 0.18	8.23^a^ ± 0.12	7.22^a^ ±0.32	7.21^a^ ± 0.15

Results are presented as mean ± standard error (*n* = 5). Means denoted with different letters in each column are significantly different.

**Table 6:** The pH and Brix of fermented coffee pulp media by different yeast species.

Strains Code	pH				Brix°			
	24 h	48 h	72 h	96 h	24 h	48 h	72 h	96 h
KNU18Y3	4.93^b^ ± 0.21	4.62^g^ ± 0.14	4.60^e^ ± 0.23	4.75^h^ ± 0.41	2.13^b^ ± 0.11	0.9^d^ ± 0.04	0.46^d^ ± 0.02	0.40^b^ ± 0.01
KNU18Y4	5.23^a^ ± 0.42	5.01^a^ ± 0.11	4.86^c^ ± 0.27	4.83^f^ ± 0.35	2.93^a^ ± 0.23	1.77^a^ ± 0.12	1.75^a^ ± 0.04	0.96^a^ ± 0.02
KNU18Y5	5.04^ab^ ± 0.12	4.97^b^ ± 0.13	5.28^b^ ± 0.17	5.86^c^ ± 0.23	2.10^b^ ± 0.14	1.66^ab^ ± 0.08	1.15^b^ ± 0.03	1.03^a^ ± 0.05
KNU18Y6	4.94^b^ ± 0.13	4.77^e^ ± 0.21	4.75^cd^ ± 0.31	4.80^g^ ± 0.24	2.06^b^ ± 0.19	1.16^cd^ ± 0.01	0.55^c^ ± 0.06	0.46^b^ ± 0.04
KNU18Y7	4.95^b^ ± 0.14	4.73^f^ ± 0.23	5.68^a^ ± 0.30	5.91^b^ ± 0.41	1.80^c^ ± 0.09	1.70^ab^ ± 0.10	1.15^b^ ± 0.07	1.01^a^ ± 0.03
KNU18Y9	4.99^b^ ± 0.15	4.95^c^ ±0.25	5.65^a^ ± 0.24	5.96^a^ ± 0.21	1.90^c^ ± 0.18	1.77^a^ ± 0.08	1.10^b^ ± 0.07	1.01^a^ ± 0.04
KNU18Y12	4.86^b^ ± 0.15	4.85^d^ ± 0.15	4.82^c^ ± 0.18	5.13^d^ ± 0.17	1.30^d^ ± 0.17	1.03^d^ ± 0.06	0.65^c^ ± 0.04	0.60^b^ ± 0.05
KNU18Y13	4.90^b^ ± 0.17	4.85^d^ ± 0.14	4.64^de^ ± 0.19	5.08^e^ ± 0.12	1.20^d^ ± 0.13	1.40^bc^ ± 0.10	0.60^c^ ± 0.08	0.36^b^ ± 0.04

Results are presented as mean ± standard error (*n* = 5). Means denoted with different letters in each column are significantly different.

**Table 7:** Pectin lyase (PL) activity of the yeasts after fermentation in CPM and SPM (24, 48, 72, and 96 h).

Strains Code	Enzymatic Activity (U mL^−1^)							
	CPM				SPM			
	24 h	48 h	72 h	96 h	24 h	48 h	72 h	96 h
KNU18Y3	16.91^b^ ± 0.16	15.44^b^ ± 0.06	8.56^ab^ ± 0.23	8.23^a^ ± 0.10	16.83^ab^ ± 0.12	15.32^b^ ± 0.40	8.41^b^ ± 0.69	7.12^a^ ± 0.23
KNU18Y4	17.66 ^a^ ± 0.13	16.25^a^ ± 0.03	8.75^a^ ± 0.04	8.24^a^ ± 0.12	17.55a ± 0.29	16.13^a^ ± 0.21	8.65^a^ ± 0.23	7.14^a^ ± 0.40
KNU18Y5	16.16 ^cd^ ± 0.12	14.68^c^ ± 0.06	8.11^d^ ± 0.08	7.75^b^ ± 0.05	16.12^abc^ ± 0.46	14.6^cd^ ± 0.12	8.98^d^ ± 0.35	7.69^bc^ ± 0.23
KNU18Y6	16.42^bcd^ ± 0.39	14.34^de^± 0.07	8.47^abc^ ± 0.06	7.32^c^ ± 0.08	16.31^abc^ ± 0.12	14.21^de^ ± 0.29	8.34^b^ ± 0.29	7.28^cde^ ± 0.40
KNU18Y7	16.45^bc^ ± 0.21	14.57^cd^ ± 0.12	8.21^cd^ ± 0.04	7.81^b^ ± 0.09	16.23^abc^ ± 0.10	14.51^cd^ ± 0.06	8.11^cd^ ± 0.49	7.76^b^ ± 0.20
KNU18Y9	16.20^cd^ ± 0.19	14.13^e^ ± 0.07	8.38^bcd^ ± 0.09	7.41^c^ ± 0.14	16.13^abc^ ± 0.29	14.09^e^ ± 0.29	8.26^bc^ ± 0.15	7.31^d^ ± 0.40
KNU18Y12	14.93^e^ ± 0.01	14.78^c^ ± 0.10	8.39^bcd^ ± 0.04	7.31^c^ ± 0.18	14.86^c^ ± 1.59	14.59^cd^ ± 0.06	8.21^bc^ ± 0.71	7.21^e^ ± 0.12
KNU18Y13	15.80^d^ ± 0.23	14.78^c^ ± 0.06	7.67 ^e^ ± 0.03	7.78^b^ ± 0.07	15.62^bc^ ± 0.23	14.66^c^ ± 0.17	7.59^e^ ± 0.23	7.64^c^ ± 0.13

Results are presented as mean ± standard error (*n* = 3). Means denoted with different letters in each column are significantly different.

**Table 8:** Polygalactrunase (PG) activity of the yeasts after fermentation in CPM and SPM (24, 48, 72, and 96 h).

Strains Code	Enzymatic Activity (U mL^−1^)							
	CPM				SPM			
	24 h	48 h	72 h	96 h	24 h	48 h	72 h	96 h
KNU18Y3	8.01^ab^ ± 0.47	7.76^b^ ± 0.12	6.08^b^ ± 0.96	6.01^c^ ± 0.05	7.98^b^ ± 0.35	7.71^b^ ± 0.23	6.02^bcd^ ± 0.17	5.91^d^ ± 0.23
KNU18Y4	8.21^a^ ± 0.50	8.28^a^ ± 0.07	8.01^a^ ± 0.11	7.75^a^ ± 0.04	8.15^a^ ± 0.29	8.21^a^ ± 0.29	7.96^a^ ± 0.29	7.71^a^ ± 0.35
KNU18Y5	7.61^abc^ ± 0.41	7.39^c^ ± 0.06	6.07^b^ ± 0.08	5.33^d^ ± 0.09	7.51^d^ ± 0.23	7.32^d^ ± 0.23	6.01^bcd^ ± 0.40	5.23^f^ ± 0.52
KNU18Y6	7.98^ab^ ± 0.47	7.68^b^ ± 0.05	5.97^b^ ± 0.18	5.88^c^ ± 0.02	7.82^c^ ± 0.46	7.61^c^ ± 0.12	5.89^d^ ± 0.52	5.7^e^ ± 0.10
KNU18Y7	6.54^cd^ ± 0.25	6.42^e^ ± 0.09	6.03^b^ ± 0.19	6.16^c^ ± 0.21	6.41^g^ ± 0.35	6.36^g^ ± 0.20	5.94^cd^ ± 0.21	6.02^c^ ± 0.23
KNU18Y9	6.98^bcd^ ± 0.32	6.77^d^ ± 0.10	6.22^b^ ± 0.05	6.59^b^ ± 0.20	6.82^e^ ± 0.29	6.67^e^ ± 0.10	6.17^bc^ ± 0.13	6.48^b^ ± 0.40
KNU18Y12	6.71^cd^ ± 0.28	6.58^de^ ± 0.09	6.02^b^ ± 0.05	6.15^c^ ± 0.15	6.68^f^ ± 0.23	6.49^f^ ± 0.40	6.21^b^ ± 0.06	6.05^c^ ± 0.23
KNU18Y13	6.06^d^ ± 0.13	5.94^f^ ± 0.10	5.75^c^ ± 0.12	5.22^d^ ± 0.14	6.03^h^ ± 0.18	5.88^h^ ± 0.35	5.75^d^ ± 0.27	5.32^f^ ± 0.32

Results are presented as mean ± standard error (*n* = 3). Means denoted with different letters within each column are significantly different.

**Table 9:** Pectin methylesterase (PME) activity of the yeasts after fermentation in CPM and SPM (24, 48, 72, and 96 h).

Strains CODE	Enzymatic Activity (U mL^−1^)							
	CPM				SPM			
	24 h	48 h	72 h	96 h	24 h	48 h	72 h	96 h
KNU18Y3	15.11^c^ ± 0.47	15.68^c^ ± 0.14	13.61^bcd^ ± 0.81	12.25^cd^ ± 0.92	15.05^b^ ± 0.35	15.56^f^ ± 0.09	13.49^bc^ ± 0.25	12.15^bc^ ± 0.13
KNU18Y4	15.23^c^ ± 0.21	15.96^bc^ ± 0.12	13.86^bc^ ± 0.92	12.56^bc^ ± 0.13	15.07^b^ ± 0.15	15.72^ef^ ± 0.12	13.71^abc^ ± 0.15	12.41^b^ ± 0.10
KNU18Y5	15.21^c^ ± 0.29	16.34^b^ ± 0.16	13.31^d^ ± 0.12	12.01^d^ ± 0.18	14.98^b^ ± 0.18	16.25^c^ ± 0.11	13.26^c^ ± 0.12	11.98^c^ ± 0.06
KNU18Y6	15.32^bc^ ± 0.24	16.07^bc^ ± 0.18	13.57^cd^ ± 0.10	12.38^bcd^ ± 0.16	15.17^b^ ± 0.19	16.02^cde^ ±0.11	13.33^c^ ± 0.07	12.19^bc^ ± 0.22
KNU18Y7	15.15^c^ ± 0.17	16.19^bc^ ± 0.20	13.56^cd^ ± 0.11	12.26^bcd^ ± 0.06	15.02^b^ ± 0.12	15.94^de^ ± 0.17	13.36^c^ ± 0.16	12.12^bc^ ± 0.12
KNU18Y9	15.23^c^± 0.08	16.3^b^ ± 0.19	13.83^bc^ ± 0.15	12.43^bc^ ± 0.08	14.98^b^ ± 0.14	16.15^cd^ ± 0.08	13.68^abc^ ± 0.09	12.27^bc^ ± 0.11
KNU18Y12	16.53^a^ ± 0.13	18.25^a^ ± 0.12	14.20^a^ ± 0.09	13.00^a^ ± 0.15	16.32^a^ ± 0.07	18.01^a^ ± 0.08	14.06^a^ ± 0.15	12.85^a^ ± 0.14
KNU18Y13	16.02^ab^ ± 0.11	17.86^a^ ± 0.23	13.90^ab^ ± 0.08	12.64^ab^ ± 0.06	15.89^a^ ± 0.16	17.63^b^ ± 0.06	13.81^ab^ ± 0.13	12.42^b^ ± 0.09

Results are presented as mean ± standard error (*n* = 3). Means denoted with different letters in each column are significantly different.
